# Hamstring muscle injury is preceded by a short period of higher running demands in professional football players

**DOI:** 10.5114/biolsport.2024.127387

**Published:** 2023-08-08

**Authors:** Victor Moreno-Perez, Víctor Sotos-Martínez, Alejandro Lopez-Valenciano, Roberto Lopez Del-Campo, Ricardo Resta, Juan Del Coso

**Affiliations:** 1 Sports Research Centre, Miguel Hernandez University of Elche, Alicante, Spain; 2 Center for Translational Research in Physiotherapy. Department of Pathology and Surgery. Miguel Hernandez University of Elche, San Joan, Spain; 3 Department of Education Science, School of Humanities and Communication Sciences, Universidad Cardenal Herrera-CEU, CEU Universities, Calle Grecia 31, 12006 Castellon de la Plana, Spain; 4 Department of Competitions, La Liga, Madrid, Spain; 5 Centre for Sport Studies, Rey Juan Carlos University, Fuenlabrada, Spain

**Keywords:** Muscle injury, Motion analysis, Match load, Soccer, Injury incidence

## Abstract

The aim of this study was to examine match running patterns before a hamstring muscle injury occurs during a match in male professional football players. A total of 281 male professional football players belonging to 7 teams from *LaLiga* were prospectively monitored over three seasons. Among these, 36 players suffered a non-contact hamstring muscle injury during an official match. The injuries were recorded by the medical staff, including the minute when the injury occurred. Running distances at different speed thresholds for 5 min and 15 min before the injury were compared to mean values of the previous 5 matches for the same time points. There were a total of 44 non-contact hamstring muscle injuries, which represents a hamstring muscle injury incidence of 3.34 injuries/1000 h of match exposure. The average time loss for these injuries was 33 ± 28 days (range 7 to 117 days). In the 15 min prior to the injury, players ran a similar distance as in control matches (*p* from 0.22 to 0.08). However, players ran a greater distance in the 5-min period before the injury than in control matches at 21.0–23.9 km/h (*p* < 0.001) and at ≥ 24 km/h (*p* < 0.001). The odds ratio for a hamstring muscle injury was 7.147 for those players who ran > 30.0 m at ≥ 21 km/h in a 5-min period (*p* < 0.001). Hamstring muscle injuries during competition were preceded by 5 min of higher running demands at > 21 km/h, compared with control matches. This suggests that a short period of unusual running increases the risk of hamstring muscle injury in professional football players.

## INTRODUCTION

Hamstring strain injury is one of the major worries in professional football [soccer] due to the high incidence and severity of this type of injury [1]. A recent study including the analysis of 2636 hamstring injuries for 21 years indicated that this type of injury represented 19% of all reported injuries [2]. Additionally, the incidence of hamstring injury has increased over time as it represented only 12% of all reported injuries in the 2001–2002 season and reached 24% of all injuries in the 2021–2022 season [2]. On average, a hamstring strain injury represents a burden of 13 days of absence although some players may need several weeks to return to play [3]. The hamstring injury incidence was 10 times higher during match play than training and around 18% of all reported hamstring injuries were recurrences with over two-thirds occurring within 2 months of the prior injury [2]. All these data indicate that hamstring injury produces a high burden for football teams with negative performance and economic implications. From a clinical viewpoint, the identification of meaningful risk factors is essential to produce effective prevention models [4]. However, the aetiology of hamstring muscle injury is complex and multifactorial and the factors that increase the likelihood of this type of injury are still under debate [4, 5].

Systematic reviews and meta-analyses conducted in football and other field-based team sports have identified several non-modifiable risk factors for hamstring muscle injuries [6–8]. Athlete’s age and the existence of a previous hamstring injury within the same season are the strongest risk factors for hamstring injury [6–8], although a previous anterior cruciate ligament injury and previous calf strain injury have also been identified as contributing factors [8]. Among the modifiable factors that may increase the likelihood of hamstring muscle injury are accumulated fatigue [9] or strength imbalances [6, 7, 10, 11], although their exact contribution to the development of the injury requires more investigation. In those players with a previous hamstring injury, there is a modification of lower limb kinematics including reductions of hip flexion and rotation [12] and a reduction of sprint performance [13] that may predispose these players to reinjury or to difficulties in returning to play. Despite the significant scientific effort made in recent years to identify the risk factors associated with hamstring injury and the implementation of prevention programmes in professional teams [14], this type of injury is still highly prevalent in professional football [3, 15]. Ekstrand et al. [2] proposed that the increase of intensity that elite men’s football has experimented with in the last years, with a larger volume of high-intensity actions and a congested competitive calendar, is a potential explanation for the high hamstring injury incidence. Hence, there is still much to understand about the causes of hamstring muscle injury, particularly about the influence of sprinting biomechanics and the effect of running exposure before the injury occurs during a match [16, 17].

As happens with all types of muscle injury in football [18, 19], the injury rates of hamstring injury are greater during matches compared with training [2, 20], likely because players are involved in higher physical demands such as larger running volumes, especially at high velocity. Recent data describing the injury-inciting events of acute hamstring injuries in professional male football players suggested that sprinting and high-intensity running are the common actions just before a hamstring injury [2, 21]. Additionally, hamstring injuries are more common in the last 15 min of each half [2, 22]. These data suggest that the combination of accumulated fatigue during a match and the need to produce a high-intensity running action to follow the play may be a trigger for hamstring injury. In this regard, two recent investigations indicate that the sprinting covered over a 1-min period [23] or a 5-min period [22] before a muscle injury occurs during a match is superior than in control matches without injury. Interestingly, when the period of running analysed is extended to 15 min before the injury, there is no difference regarding control matches. These investigations suggest that a 1-to-5 min period of higher-than-habitual intense running increases the odds of muscle injury in professional football players. However, there is no information to determine whether a period of high-intensity running above the normal is also an inciting event of acute hamstring injury. The aim of this study was to examine match running patterns before a hamstring muscle injury occurs during a match in male professional football players.

## MATERIALS AND METHODS

### Participants

A total of 281 male professional football players of 7 teams competing in the top division of the Spanish football league (*LaLiga*) were prospectively monitored over three seasons (2016–2017, 2017–2018 and 2018–2019). This was a convenience sample as we obtained data from the medical staff of professional football teams that had collaborated with our research team in previous investigations. From this sample, only 36 players suffered a hamstring muscle injury, and they were selected for the current investigation (age: 26.5 ± 4.2 years; body mass: 72.4 ± 5.4 kg; height: 179.2 ± 5.8 cm). In the final sample, there were 8 external midfielders, 8 external defenders, 10 forwards, 3 central midfielders and 7 central defenders. All players performed ~ 8–12 hours of football training and 1–2 competitive matches per week. Before the start of this investigation, an institutional Ethics Review Committee (code: DPC.VMP.01.18) approved the procedures included used in this study, following the latest version of the Declaration of Helsinki. In addition, *LaLiga* has authorized the use of data on players’ running performance during official matches and the study does not contain information to identify players, as per *LaLiga’s* ethical guidelines for research.

### Injury data collection

In each team, non-contact hamstring muscle injuries were diagnosed and recorded by the medical staff using the classification system developed by the International Olympic Committee Consensus Group [24]. To be included in the study, the hamstring injury should be characterized by representing a player’s physical complaint during an official competitive match, which prevented the player from participating in the following competition or training session [25]. Only hamstring injuries that produced an absence of at least seven days were considered for this investigation to avoid the influence of minor complaints [21]. All injuries were confirmed by magnetic resonance imaging (MRI). The injury report included the minute when the injury occurred during the match (certified by video analysis), injury severity (number of days from the date of injury to the date of returning to full participation), and the existence of recurrence (an injury of the same type and at the same site). An injury of the same type and at the same site occurring previously during the same season was catalogued as recurrent injury. All injuries were recorded in an electronic document which was ended once the player returned to play. Ended injury reports were forwarded to the research group once a month during the duration of the study and the researchers included injury data in an *ad hoc* database.

### Data collection of running patterns during match play

Match performance data were collected using a validated multicamera tracking system called Mediacoach [26, 27]. Mediacoach consists of 8 stable synchronized and calibrated cameras positioned at the top of the stadium with a sampling frequency of 25 Hz. Mediacoach also contains associated software to analyse running demands and match events for each player. Running data of the players who sustained a hamstring muscle injury during the match were analysed for 15 minutes and 5 minutes before the injury event, according to the injury report. The moment of injury was confirmed by video analysis. To be valid, the player had to be a starter player in the match where the injury occurred. Running data before the injury were compared with normative data of the same player in the previous 5 matches, mimicking the duration of the periods and the minutes analysed (i.e., control situation). To be valid, the 5 “control” matches had to be carried out within the 30 days before the injury and the player had to be a starter player, to mimic the conditions of the “injury” match. The following physical performance variables were selected for analysis: the total distance covered (m), the distance at 18.0–20.9 km/h (intense running), the distance at 21.0–23.9 km/h (sprinting at low intensity) and the distance at ≥ 24.0 km/h (sprinting at high intensity), following a previous classification of running speed thresholds in professional football [28]. Running distance covered at ≥ 21.0 km/h was also calculated to identify the sprint distance. Additionally, information about match venue (home or away match) and match result (win, draw, or loss) was obtained in all matches where a hamstring muscle injury occurred.

### Statistical analysis

The normality of the data was verified using the Shapiro-Wilk test. All variables had a normal distribution (p > 0.050). Data are presented as frequencies for qualitative variables and as mean ± SD for quantitative variables. Differences in the distribution of injuries depending on the match day, match time and severity were identified with χ^2^ tests and standardized residuals. A two-way ANOVA (running speed × injury; 3 × 2) was used to compare running distances at different velocities in the match with injury with respect to control matches. The sphericity assumption was checked with Mauchly’s test. ANOVA effect sizes were calculated with partial eta squared (ηp^2^) and interpreted as follows: small, 0.02; medium, 0.13; large, 0.26. In the case of running velocity, injury, or interaction between these two factors, pairwise comparisons between data of the match with injury and control matches were made with Bonferroni *post hoc* tests. Paired-samples t-tests were used to compare total distance and distance at ≥ 21 km/h in a match with hamstring muscle injury vs control matches. These analyses were performed separately for 5- and 15-min periods. The level of statistical significance for the differences was set at *p* < 0.050. Cohen’s *d* and its 95% confidence intervals (CI) were calculated as effect size (ES) for those variables with a statistically significant difference and interpreted as < 0.2, trivial; 0.2–0.6, small; 0.6–1.2, moderate; 1.2–2.0, large; 2.0–4.0, very large and; > 4.0, extremely large. The odds ratio (OR) and 95% CI were calculated for running distance at ≥ 21 km/h for the 5 min before the injury. All analyses were performed using the SPSS software (version 27.0; IBM, Armonk, NY, USA).

## RESULTS

**FIG. 1:**
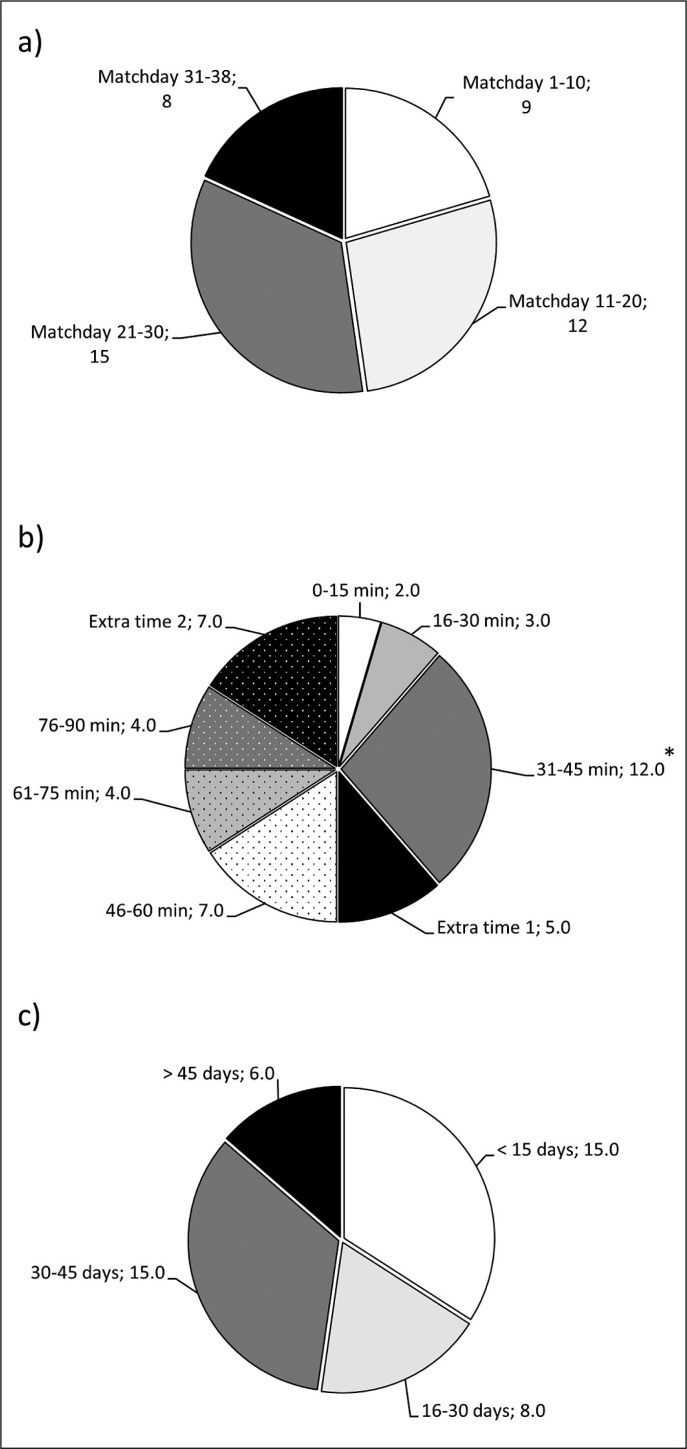
Proportion of non-contact hamstring muscle injuries that occurred during professional football competition according to match day (a), minute (b) and (c) severity. Data in the pie charts are proportions and the number of injuries within each category is included in the label of the category (*) Different from the expected value at *p* < 0.05.

A total of 44 non-contact hamstring muscle injuries occurred during official matches for the three seasons under investigation. This represented an overall hamstring muscle injury incidence of 3.34 injuries per 1000 h of match exposure (range = 0.0–17.5 hamstring injuries per 1000 h of match exposure). Thirty players had only one hamstring muscle injury, four players had two muscle injuries and two players had three muscle injuries during the study period. From the total, 18.2% of the hamstring injuries recorded were catalogued as recurrent injury. The injuries were equally distributed across all the season (*p* = 0.304, Figure 1a) but there was a higher number of injuries in the last 15 min of the first half when compared to other 15-min sections of the matches (*p* < 0.05, Figure 1b). The average time loss for these injuries was 33 ± 28 days (range 7 to 117 days) with 15% of injuries needing more than 45 days of recovery (Figure 1c). There was an equal distribution of injuries during matches played at home (54.5%) and away (45.4%; *p* = 0.394). The result of the match at the moment of injury was win/draw/lose = 50.0/20.5/29.5%) for the team of the injured player, which was similar to the control matches (*p* = 0.168).

**FIG. 2:**
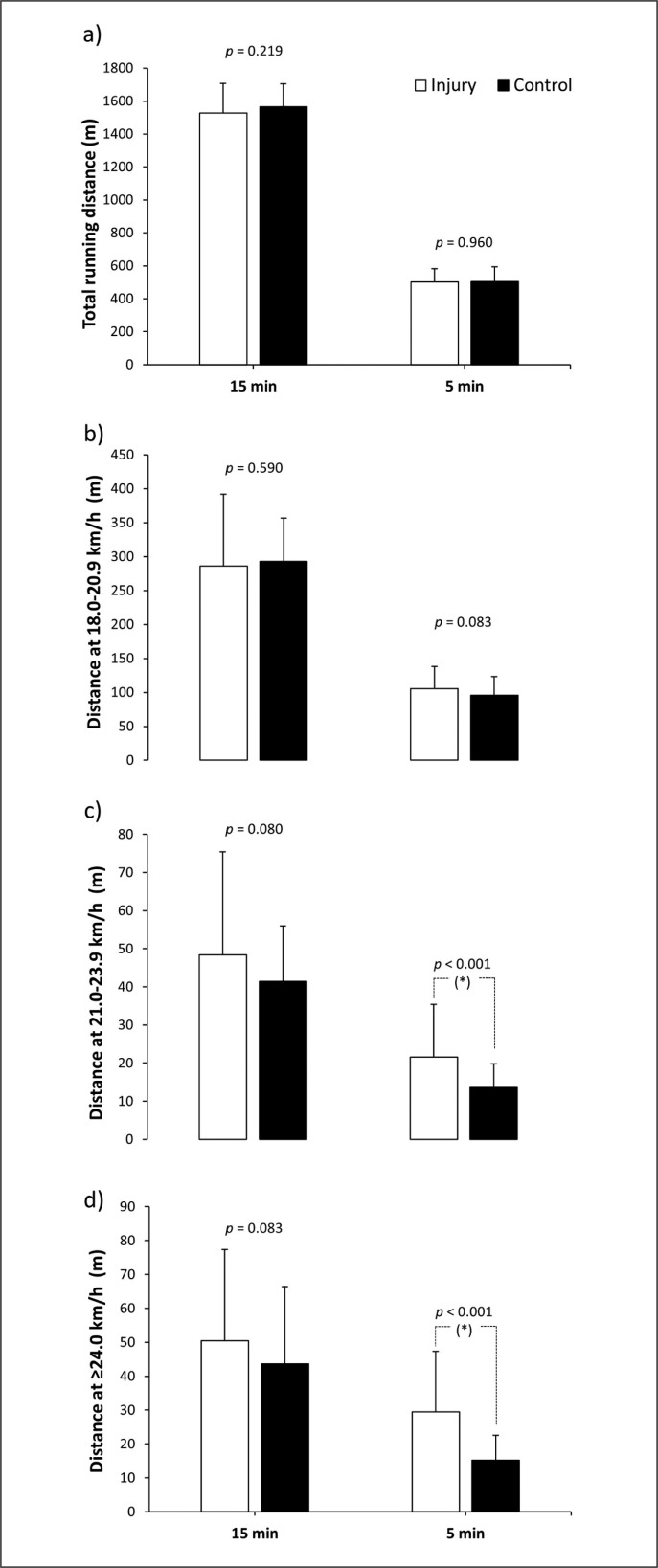
Total running distance (a), running distance at 18.0–20.9 km/h (b), at 21.0–23.9 km/h (c), and ≥ 24 km/h (c) in the 15 and 5 min prior to suffering a non-contact hamstring muscle injury compared to the same match period of control matches. “Injury” refers to running distances at different velocities in the match with injury. “Control” refers to running distances at different velocities in the previous 5 matches for the same time points. (*) Statistically significant difference between control and injury condition at *p* < 0.05.

Figure 2 contains data on total running distance and distance at different speed thresholds for 15-min and 5-min periods before the injury occurred compared to the same periods of time in the control matches without injury. In the 15-min period prior to the injury, total running distance was similar to control matches (*p* = 0.219). Regarding the analysis of running distance at different speed thresholds, there was a main effect of the running speed on the running distance covered (F = 496.10, *p* < 0.001, ηp^2^ = 0.920), but there was no main effect of injury (F = 0.16, *p* = 0.692, ηp^2^ = 0.004) or running speed × injury interaction (F = 1.57, *p* = 0.570, ηp^2^ = 0.024). The *post hoc* analysis indicated that players ran a similar distance 15 min before the injury compared with the control matches (*p* from 0.590 to 0.083). In the 5-min period prior to the injury, total running distance was similar to control matches (*p* = 0.960). However, there were main effects of the running speed (F = 460.47, *p* < 0.001, ηp^2^ = 0.915) and of the injury (F = 22.67, *p* < 0.001, ηp^2^ = 0.345) on the running speed covered at different speed thresholds, with no running speed × injury interaction (F = 0.80, *p* = 0.451, ηp^2^ = 0.018). The *post hoc* analysis indicated that players ran a greater distance in the 5-min period before the injury compared with the control matches at 21.0–23.9 km/h (*p* < 0.001; ES = 0.57 [0.23, 0.89]) and at ≥ 24 km/h (*p* < 0.001; ES = 0.78 [0.43, 1.12]), with no difference at 18.0–20.9 km/h (*p* = 0.083).

**FIG. 3:**
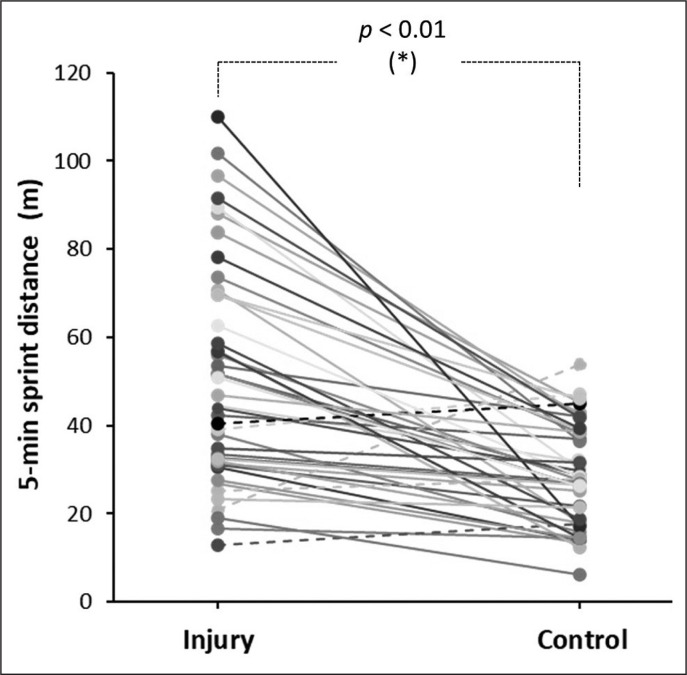
Running distance at ≥ 21 km/h 5 min prior to suffering a non-contact hamstring muscle injury compared to the same match period of control matches. Each line represents individual values for 5 min before the injury and the same period of control matches. Continuous lines depict participants with higher values on the day of injury than in the control matches and the dashed lines depict participants with lower values on the day of injury than in the control matches. (*) Statistically significant difference between control and injury condition at *p* < 0.05.

Figure 3 contains individual data for the running distance at ≥ 21 km/h in the 5 min before suffering a non-contact hamstring muscle injury compared to the same match period of control matches. Overall, the running distance at ≥ 21 km/h was greater on the day of the injury than in the control matches (51.1 ± 24.7 vs 28.8 ± 11.5 m; *p* < 0.001; ES = 1.09 [0.70, 1.47]). Of the 44 injuries, 39 (88.6%) of them were preceded by a 5-min period of greater running distance at ≥ 21 km/h. The OR for a hamstring muscle injury was 7.147 (95% CI = 2.689 to 18.992) for those players who ran > 30.0 m at ≥ 21 km/h in a 5-min period (*p* < 0.001).

## DISCUSSION

The aim of this study was to examine match running patterns before a hamstring muscle injury occurs during a match in male professional football players. This investigation was designed to determine whether a period of more intense than usual running is an inciting event for acute hamstring injuries in professional football. The main result of the study was that players showed an increase of the distance covered running at high intensity (21–24 km/h; +76.1 ± 110.0%) and at sprint velocity (> 24 km/h; +114.0 ± 152.3%) in the 5 minutes before the injury occurred when compared to the same periods of five control matches (Figure 2). This scenario of unusual high-intensity running was present in most injury events as 39 out of 44 hamstring muscle injuries were preceded by a 5-min period of high-intensity running (i.e., distance covered at ≥ 21 km/h) of higher intensity than the high-intensity running performed in the control matches (Figure 3). Interestingly, the total distance and the distance covered at 18.0–20.9 km/h in the 5 minutes before the injury were similar to the control matches. Collectively, this information suggests that a short period of higher-than-usual sprint-like distance may trigger the onset of a hamstring muscle injury. Specifically, sprinting more than 30 m for a period of 5 min may increase the likelihood of hamstring injury by several-fold.

Muscle injury is a complex phenomenon determined by the non-linear interaction of several factors, but fatigue and high external work-loads are within the most common inciting factors for this type of injury [29]. Similar findings when analysing all types of muscle injuries have been previously reported in professional football players competing in the Qatar Stars League [23] and *LaLiga* [22]. Specifically, Gregson et al. [23] investigated running distances at different speed thresholds 1 min and 5 min prior to a muscle injury in comparison to normative running data and they found that an increase in sprinting distance of 11 m, covered over a 1-min period, increased the odds of muscle injury. Moreno-Perez et al. [22] carried out a similar investigation but they analysed running distances 15 min and 5 min prior to the muscle injury. These authors found that the running distance covered at all intensities was similar for the 15 min before the muscle injury as in control matches, but the distance covered at sprinting was greater for the 5 min before the injury event. The current investigation complements these outcomes as it indicates that an unusually long sprinting distance covered during a short period may trigger the onset of hamstring muscle injury. It has been recently reported by video analysis that rapid movements with high eccentric demands of the posterior thigh are likely the main hamstring injury mechanism [21]. These movements include linear acceleration, high-intensity running, closed chain movements such as braking or stopping and open chain movements such as kicking. These inciting events are more likely associated with a hamstring strain when they are preceded by a short period of high-intensity running, as this and previous investigations have reported [22, 23]. Overall, all these studies contribute to understanding how hamstring injuries occur in professional football and support the need to create custom prevention programmes for hamstring muscle injury that include short periods of high-intensity running, mimicking the demands of the game and the performance of rapid movements with high eccentric demands of the posterior thigh in a controlled state of fatigue.

In line with previous research [2, 30], the current findings confirm that the number of hamstring injuries increases at the end of the first half of the match (Figure 1). Interestingly, the proportion of injuries in extra time 1 and extra time 2 was similar to the other 15-min periods despite extra times being on average ~1.6 min and ~4.2 min for the matches with injury. This indirectly suggests a high rate of injury during extra times, particularly in the second half. A possible explanation for the observed results may involve the interactions between acute fatigue and hamstring muscle activation and function [9, 31]. It is well known that a competitive football match leads to acute fatigue and a reduction of performance that normally occurs at the end of the first half, and with more severity at the end of the match [32, 33]. The fatigue developed during a football match is probably linked to the depletion of muscle glycogen concentrations [34] and entails a decline in physical performance [35, 36], altered coordination of muscle activation patterns [9] and changes in lower limb kinematics [37]. Additionally, muscle fatigue may alter the neuromuscular coordination and cause an excessive tensile load on adjacent tissues, decreased hip flexion, increased knee extension and anterior pelvic tilt [37]. Recent data indicate that there is a reduced capacity of the hamstrings to decelerate the lower leg during sprint running with fatigue [31]. Taken together, all this information explains why hamstring muscle injury is more likely to occur at the end of each half [2], as players are more fatigued and running kinematics is modified. Hence, fatigue and all the above-mentioned fatigue-induced changes in performance, running kinematics and neuromuscular coordination can be considered as potential contributors to hamstring injury, at least during competitive matches. However, further investigation is required to determine to what degree fatigue is a mechanism of hamstring injury in professional football. In this regard, the independent and combined analysis of the effects of acute fatigue (transient and developed during the match) and the chronic residual fatigue, developed over the season as result of the competitive calendar and the training workload [38], on hamstring injury incidence should be developed in future investigations.

Several potential limitations may be recognized in the present study. Firstly, as the current study has been performed on a sample of male professional football players, the findings may not be generalizable to other professional team sport athletes or female football players. Further, the present study did not quantify the training load carried out during the training week before the matches where the injury occurred. Probably, the occurrence of the hamstring muscle injury was also associated with a higher training workload and higher self-perceived fatigue in those players who suffered the injury, as these two factors have been found in the week prior to a muscle injury in professional football players [22]. Additionally, the cumulative training workload for several weeks can also increase the risk of muscle injury [39]. Therefore, future studies could benefit from investigating the internal and external loads in the days and weeks before the hamstring injury to examine the contribution of these factors to hamstring muscle injury aetiology. The current study only included the analysis of running 5 and 15 min before the injury occurred. However, it is possible that the effect of prior fatigue accumulated throughout the match also contributed to the mechanism of hamstring injury. Future investigations should include the analysis of running patterns for longer periods before the injury occurs during a match. Only hamstring muscle injuries during competitive matches in male professionals were investigated while hamstring strains during training may have a different mechanism of onset. Last, 8 out of the 44 hamstring injuries analysed in this investigation were classified as recurrent injury, which represents 18.2% of the total number of injuries, a value similar to previous data [2, 3]. However, this low number of recurrent injuries impeded performance of a sub-analysis of running patterns before injury vs recurrent injuries with proper statistical power.

## CONCLUSIONS

Male professional football players who suffered a non-contact hamstring muscle injury during a competitive match ran a greater distance at ≥ 21 km/h in the 5 minutes before the injury, at least when compared to their habitual ≥ 21 km/h running distance at the same match time in control matches without injury. Additionally, a high proportion of injuries occurred in the last 15 min of each half, suggesting a potential role of high-intensity running and accumulated fatigue in the development of hamstring muscle injuries. These results could be used to create specific training programmes and recovery strategies which can help to prepare players for periods of higher-than-habitual running demands during competitive matches, ultimately decreasing the incidence of hamstring muscle injuries in professional football.

In summary, this study reveals that hamstring muscle injuries during competition were preceded in most cases by 5 min of higher running demands at > 21 km/h, compared with the five prior matches. This outcome suggests that a short period of unusual running at high speed increases the risk of hamstring muscle injury in professional football players.
